# Our Voluntary Hospitals

**Published:** 1903-05-09

**Authors:** 


### The Hospital.

A Journal of

The Medical Sciences and Hospital Administration.

Edited by Sir Henry Burdett, K.C.B.

Vol. XXXIV.—No. 867. [May 9, 1903.

### Our Voluntary Hospitals.

Mr. J. G. Craggs has published through the Scientific Press a brochure entitled "Rate-Supported *v.* Voluntary Hospitals." This is an interesting compilation which deserves, and will no doubt receive, the attention of all who are interested in hospitals especially, and also of the thinking portion of the general public. We are all very much indebted to Mr. Craggs for the labour he has expended upon its compilation, and we feel it will usefully draw attention to certain aspects of the hospital question upon which misapprehension prevails at present.

Mr. Craggs first deals with hospitals supported by rates, but, owing to the absence of accurate data, he has confined his remarks mainly to the institutions under the control of the Metropolitan Asylums Board. He properly praises that Board for the complete statistics they issue, but points out that to be really effective a modification is called for in the direction of closing the financial accounts and the medical statistical year at Lady-Day or Michaelmas. Until the medical and financial years end on the same day, the statistics issued by the Asylums Board can never be available for comparative purposes. The circumstances which surround the provision of hospital accommodation for epidemic and infectious diseases differ materially from those relating to the general hospitals of a great city like London. It is not possible to draw helpful or accurate conclusions from the relative cost of maintaining a patient in these two types of hospitals. What is possible is to ascertain the actual cost of buildings, sites, and equipment for the two classes of hospitals, and from the tables thus compiled to deduce facts, which must have a material bearing for good upon the cost of hospital construction in the near future. We hope that Mr. Craggs will devote his attention to this branch of the subject, and that in a second edition it may be possible for him to bring together an array of accurate figures with this object.

The main purpose which Mr. Craggs appears to have in view is, however, to show, that for economical reasons especially, it is to the interests of all classes of the community, more particularly of the owners and occupiers of fine blocks of buildings in a city like London, that the latter should contribute voluntarily a definite sum per annum to the maintenance of the voluntary hospitals. During the last 20 years, especially, the tendency in the metropolis has been for private firms to give place to companies, established under the Limited Liability Acts. Mr. Craggs insists, that all the more important undertakings worked as limited liability companies, or under other corporate forms, especially banks and insurance companies, railway companies, and so forth, should, in their own interest and for their own advantage, contribute in their corporate capacity a reasonable annual sum, to preserve and carry on the London hospitals. Meeting the feeling among the directors of such undertakings that they are trustees for the shareholders and so are not justified in making such a subscription for the purposes named, Mr. Craggs expresses sympathy as a professional auditor with this view, except, as in the case of the hospitals, where the gifts go to institutions which may be regarded as auxiliary to the welfare of each of these companies, and so the directors are bound in the interests of their shareholders, to protect them against new rates and taxes by contributing to the existing hospital system. We shall rejoice if Mr. Craggs' crusade proves successful, and so our hospitals may be once more enabled to rely upon generous contributions from all the leading enterprises which have their centre in the city of London.

Whatever may be the general opinion of directors of miscellaneous companies, Mr. Craggs seems to us to make a good case against the life insurance companies, which he points out, are greatly assisted in profit making by the medical skill and knowledge resulting from the hospitals of London, and in many cases by the direct services of the hospitals to the persons insured. Many of the original donors to hospitals lived before the days of limited liability, and carried on considerable businesses which it is the present fashion for a company to conduct. The private owners recognised their duties as citizens, and it is to the public interest, that the companies, which have followed in their steps as money-making concerns, should likewise fulfil their duties as corporate burgesses. No company can claim, because it is limited, nor can its directors claim because they are trustees for others, that they shall not pay rates and taxes out of the corporate funds. Mr. Craggs further contends, that they cannot justly omit to pay a reasonable quota to the voluntary hospitals, as a kind of insurance against greatly increased taxation in the near future, which otherwise must entail the payment of heavy rates for the maintenance of an adequate hospital system. Add to this the knowledge which Mr. Craggs' figures yield, that the voluntary system of hospital maintenance is far the most economical; that it is attended by material advantages in regard to both scientific development and efficient medical treatment which might otherwise be wanting; and we have surely said enough to excite the interest of the majority of Londoners in these questions, and to secure the candid examination and sympathetic study of the points raised by Mr. Craggs in the brochure before us.

